# Supplementary figures and images for: Selected Plant-Derived Polyphenols as Potential Therapeutic Agents for Peripheral Artery Disease: Molecular Mechanisms, Efficacy and Safety
Source: Molecules. 2022 Oct 21;27(20):7110. doi: 10.3390/molecules27207110 (PMC9611444; doi:10.3390/molecules27207110)

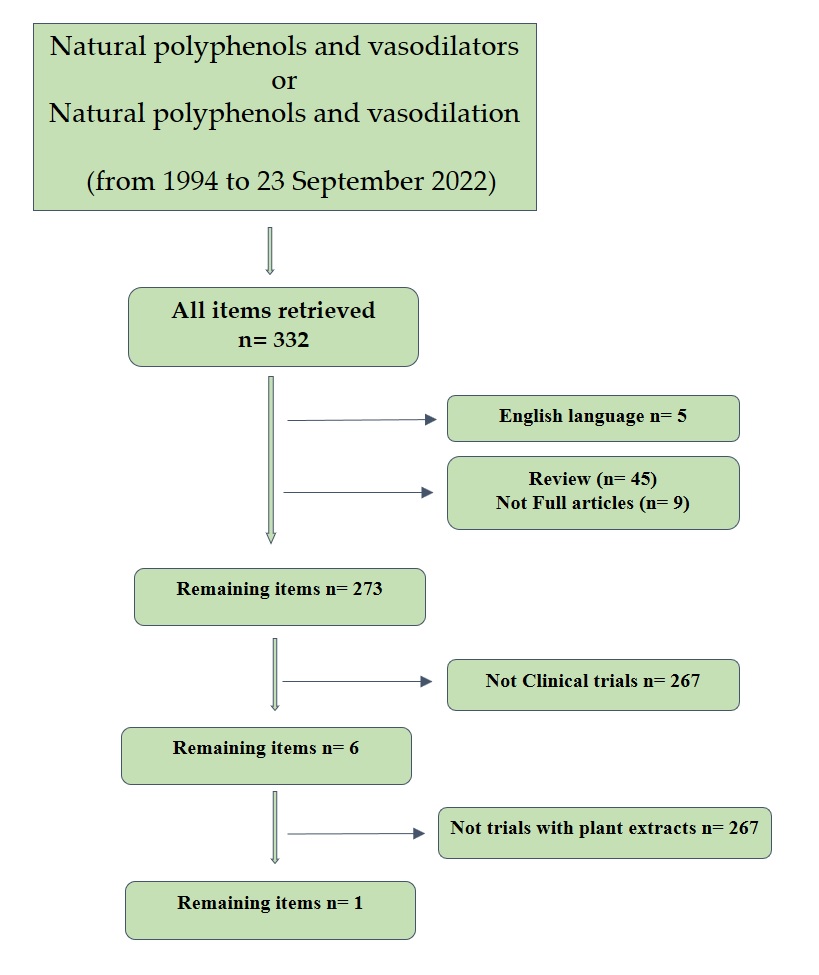

Supplement: Supplementary file 1 [file molecules-27-07110-s001.zip › Figure S1 Flowchart Molecules Septembre 2022.jpg]

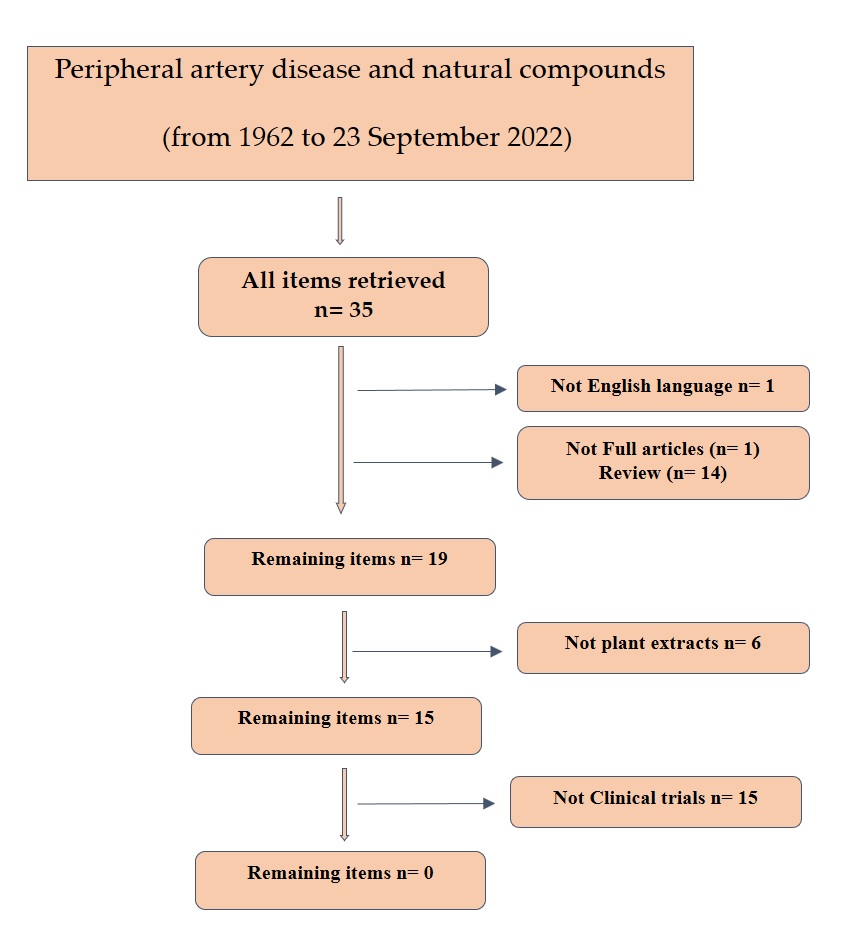

Supplement: Supplementary file 1 [file molecules-27-07110-s001.zip › Figure S2 Flowchart Review Molecules September 2022.jpg]
